# Editorial: Medicine and food homology: emerging tool and methodology for separation and analysis of the bioactive factors

**DOI:** 10.3389/fnut.2023.1288237

**Published:** 2023-10-11

**Authors:** Yisheng Chen, Wenyi Kang, Irena M. Choma, Haining Zhuang

**Affiliations:** ^1^College of Food Science and Engineering, Shanxi Agricultural University, Taiyuan, China; ^2^National R&D Center for Edible Fungus Processing Technology, Henan University, Zhengzhou, China; ^3^Department of Chromatography, Institute of Chemical Sciences, Faculty of Chemistry, Marie Curie-Skłodowska University, Lublin, Poland; ^4^School of Food and Tourism, Shanghai Urban Construction Vocational College, Shanghai, China

**Keywords:** medicine and food homology, bioactive factor, separation, functional value, analytical tool

This Research Topic was dedicated to introducing emerging tool and methodology tailored for dealing with separation, identification, authentication, and elucidation of bioactive factors in Medicine and Food Homology (MFH) materials and products, in a direct and cost-efficient way. From the manuscripts submitted to the editorial team, a total of eight original research article and one review article focusing on this theme were eventually selected for publication.

With the increasing awareness of healthy life and scientific nutrition, the conception of MFH attracted marked attention around the world. MFH refers to edible materials that can be both employed as food and medicines. As matter of fact, there is no absolute boundary between each other. As the critical intersection of food and medicine, MFH materials are not only rich in nutrition, but also able to maintain people health, prevent, and cure diseases. With a long-standing history around the world, the revival and popularity of MFH conform to today's public trends of returning to a natural and healthy life. More importantly, MFH materials are a treasure resource of bioactive factors for current health-beneficial food and pharmaceuticals. Instead of consuming them in the form of raw material, future direction of MFH research is to unambiguously identify the compound responsible for the bioactivity, precisely isolate these bioactive factors and apply them into value-added usage, making them into more widely accepted functional foods and therapeutic drugs.

Therefore, further excavation and better understanding of these bioactive factors would be greatly helpful for sparkling innovative ideas of MFH materials processing. Nevertheless, the MFH material, as a whole, is a rather sophisticated bio-mixture, containing large array of phytochemicals. Besides, functional value of the MFH materials is highly influenced by many factors. More specifically, the real efficacy component may be flooded by the huge number of co-existed phytochemicals. Moreover, conventional analytical approaches are not able to directly and accurately establish the relationship between chemical structures and observed bioactivity. All these difficulties pose serious challenges to the precise separation, identification, authentication, and elucidation of bioactive factors in MFH materials. As a key to solve these bottlenecks, powerful tool and methodology are urgently needed.

The research targets of the nine articles presented in this Research Topic covered a large array of MFH samples, including *Rehmannia glutinosa, Dendrobium officinale*, Cistanche, Mung Beans, *Rugosoannulata*, Poria, and *Tremella aurantialba*, vegetable oil. The individual bioactive factor, such as unsaturated fatty acid, polyphenol, oligosaccharide, and peptide, mainly responsible for the observed bioactivity in these MFH was individually separated and evaluated, chemically, biologically, and pharmacologically. A couple of state-of-the-art analytical tools and methodologies were highlighted in these articles.

Generally, the importance of ultra-high-performance liquid chromatography (UHPLC) was acknowledge in the separation of MFH, enabling simultaneous separation of hundreds of chemicals in MFH. On this basis, structural elucidation of the obtained separation result can be easily realized with the aid of advanced mass spectrometers, which is greatly helpful for the targeted and un-targeted analysis of MFH. Remarkably, a series of specialized software and online-database was introduced and exemplarily studied in this Research Topic. In combination with advanced separation devices, these powerful tools opened a new horizon for precisely matching the bioactivity of compounds with targeted biomacromolecules. For instance, Yan et al. attempted to employ network pharmacological analysis for unveiling the pharmacological mechanism of *Tremella aurantialba*, in which compounds with potential therapeutic effect for nervous system, immune system, endocrine system, neoplasm system, as well as cardiovascular system diseases were virtually identified. Guided by the similar principle, spectrum-effect, component knock-out, and molecular docking technique were used by Ma et al. for the analysis of Poria, unambiguously confirm that eight individual compounds, i.e., poricoic acid B, dehydrotumulosic acid, poricoic acid A, polyporenic acid C, 3- epidehydrotumulosic acid, dehydropachymic acid, 3-O-acetyl-16α-hydroxytrametenolic acid, and pachymic acid, were mainly responsible for inhibitory activity against α-glucosidase. These studies could be good examples showing how to establish the relationship between the observed bioactivity and the related compound in MFH.

Apart from column chromatographic tools like HPLC/GC utilized in the articles within this Research Topic, the editorial team was very enthusiastic to introduce the innovative application of instrumentalized planar chromatography (the so called high performance thin-layer chromatography, HPTLC) as a flexible and versatile tool for MFH analysis. Apart from being able to perform 2-dimentional chromatographic separation, HPTLC was more importantly used by Müller et al. as an all-in-one platform efficiently combining on-surface metabolization and multi-bioassays for profiling the real health function of vegetable oils. Particularly, this analytical tool can be performed without elaborate sample preparation or fractionation to ensure sample integrity. Thus, no sample part was lost, and the whole sample was studied on a single surface regarding all aspects. This made the methodology as well as technology miniaturized, lean, all-in-one, and very sustainable for screening bioactivity of MFH. This study may be a good evidence that instrumentalized planar chromatography is still a essential tool for MFH analysis.

All in all, the nine articles worth being read.

## Author contributions

YC: Conceptualization, Data curation, Funding acquisition, Writing—original draft, Writing—review and editing. WK: Writing—review and editing. IC: Writing—review and editing. HZ: Writing—review and editing.

